# Prognostic or predictive value of circulating cytokines and angiogenic factors for initial treatment of multiple myeloma in the GIMEMA MM0305 randomized controlled trial

**DOI:** 10.1186/s13045-018-0691-4

**Published:** 2019-01-09

**Authors:** Ilaria Saltarella, Fortunato Morabito, Nicola Giuliani, Carolina Terragna, Paola Omedè, Antonio Palumbo, Sara Bringhen, Lorenzo De Paoli, Enrica Martino, Alessandra Larocca, Massimo Offidani, Francesca Patriarca, Chiara Nozzoli, Tommasina Guglielmelli, Giulia Benevolo, Vincenzo Callea, Luca Baldini, Mariella Grasso, Giovanna Leonardi, Manuela Rizzo, Antonietta Pia Falcone, Daniela Gottardi, Vittorio Montefusco, Pellegrino Musto, Maria Teresa Petrucci, Franco Dammacco, Mario Boccadoro, Angelo Vacca, Roberto Ria

**Affiliations:** 1 Department of Biomedical Science and Human Oncology, University of Bari “Aldo Moro” Medical School, Bari, Italy; 2 Biothecnology Research Unit, Aprigliano, Cosenza Italy; 3 Hemato-oncology Department Augusta Victoria Hospital, Jerusalem, Israel; 4 Department of Clinical and Experimental Medicine, Myeloma Unit, University of Parma, Parma, Italy; 5 “Seràgnoli” Institute of Hematology, Department of Experimental, Diagnostic and Specialty Medicine (DIMES), Bologna University School of Medicine, Bologna, Italy; 6 Division of Hematology, University of Torino, Azienda Ospedaliero-Universitaria Città della Salute e della Scienza di Torino, Turin, Italy; 7 Università degli Studi del Piemonte Orientale, Novara, Italy; 8 Division of Hematology, AOU “Policlinico-Vittorio Emanuele”, Catania, Italy; 9 Hematology Unit A.O.U. Ospedali Riuniti, Ancona, Italy; 10 DAME, University of Udine, Udine, Italy; 11 Cellular Therapies and Transfusion Medicine Unit, AOU Careggi, Florence, Italy; 12 A.O. San Luigi, Gonzaga, Orbassano, Turin, Italy; 13 SC Hematology AO Città della Salute e della Scienza, Turin, Italy; 14 Divisione di Ematologia, Ospedali Riuniti, Reggio di Calabria, Italy; 15 Hematology Unit, Fondazione IRCCS, Cà Granda, OM Policlinico, DIPO, University of Milan, Milan, Italy; 16 A.O. S. Croce e Carle, Cuneo, Italy; 17 Department of Oncology and Hematology AOU, Hematology Unit, Modena, Italy; 18 University Tor Vergata, Rome, Italy; 19 IRCCS Casa Sollievo della Sofferenza, San Giovanni Rotondo, Italy; 20 A.O.U. S. Giovanni Battista A.O. Mauriziano-Umberto I, Turin, Italy; 21 IRCCS Istituto Nazionale per lo Studio e la Cura dei Tumori, Milan, Italy; 22 IRCCS Centro di Riferimento Oncologico della Basilicata, Rionero in Vulture, Italy; 23 Hematology, Department of Cellular Biotechnologies and Hematology, Sapienza University of Rome, Rome, Italy; 24 Internal Medicine “G. Baccelli”, Myeloma Unit, University of Bari “Aldo Moro” Medical School, Azienda Ospedaliero-Universitaria Policlinico, Piazza Giulio Cesare 11, 70124 Bari, Italy

**Keywords:** Angiogenic factors, Multiple myeloma, Overall survival, Progression-free survival, Response rate

## Abstract

**Background:**

Several new drugs are approved for treatment of patients with multiple myeloma (MM), but no validated biomarkers are available for the prediction of a clinical outcome. We aimed to establish whether pretreatment blood and bone marrow plasma concentrations of major cytokines and angiogenic factors (CAFs) of patients from a phase 3 trial of a MM treatment could have a prognostic and predictive value in terms of response to therapy and progression-free and overall survival and whether these patients could be stratified for their prognosis.

**Methods:**

Blood and bone marrow plasma levels of Ang-2, FGF-2, HGF, VEGF, PDGF-β, IL-8, TNF-α, TIMP-1, and TIMP-2 were determined at diagnosis in MM patients enrolled in the GIMEMA MM0305 randomized controlled trial by an enzyme-linked immunosorbent assay (ELISA). These levels were correlated both reciprocally and with the type of therapy and patients’ characteristics and with a group of non-MM patients as controls.

**Results:**

No significant differences were detected between the blood and bone marrow plasma levels of angiogenic cytokines. A cutoff for each CAF was established. The therapeutic response of patients with blood plasma levels of CAFs lower than the cutoff was better than the response of those with higher levels in terms of percentage of responding patients and quality of response.

**Conclusion:**

FGF-2, HGF, VEGF, and PDGF-β plasma levels at diagnosis have predictive significance for response to treatment. The stratification of patients based on the levels of CAFs at diagnosis and their variations after therapy is useful to characterize different risk groups concerning outcome and response to therapy.

**Trial registration:**

Clinical trial information can be found at the following link: NCT01063179

**Electronic supplementary material:**

The online version of this article (10.1186/s13045-018-0691-4) contains supplementary material, which is available to authorized users.

## Background

Multiple myeloma (MM) is the second most common hematologic cancer after non-Hodgkin’s lymphoma, with a higher incidence in the elderly. Patients older than 70 years account for 56% of new cases and for 73% of all deaths from MM [1]. Combined melphalan-prednisone has been the standard of care for more than 40 years and has been found to be associated with a median survival of 29 to 37 months [2–4]. Today, the availability of novel agents, such as the first-in-class proteasome inhibitor bortezomib and the immunomodulatory drugs thalidomide and lenalidomide, has significantly improved the clinical outcome of these patients [5–13].

Accurate identification of high-risk patients and risk stratification are crucial in improving outcomes for MM patients, but considerable heterogeneity exists in their overall survival. Although a large number of prognostic markers have been described, including disease burden (Durie-Salmon staging system, International Staging System, magnetic resonance imaging, (18F)fluorodeoxyglucose positron emission tomography, presence of extramedullary disease or plasma-cell leukemia), host factors (age, performance status, and renal function), tumor biology (proliferation rate, conventional cytogenetics, interphase fluorescence in situ hybridization, and gene expression profiling), and depth of response to therapy [14–18], none of them completely explains the heterogeneity seen in this tumor.

To further complicate matters, some of the new treatments appear to overcome the high risk defined by one or more of these prognostic factors [19, 20]. With the increased treatment options, the ability of some treatments to overcome certain risk factors, and the availability of markers to define risk categories, risk stratification in the management of MM is becoming an important issue [21]. The achievement of a uniform risk stratification system would also allow a better comparison of patient groups across different trials [22].

Angiogenesis is a constant hallmark of MM progression and has prognostic potential. The pathophysiology of MM-induced angiogenesis involves both direct production of angiogenic cytokines by plasma cells and their induction within the bone marrow microenvironment [23]. Moreover, inhibitors of the vascular endothelial growth factor (VEGF) pathway, including bevacizumab and tyrosine kinase inhibitors such as sorafenib, sunitinib, and pazopanib, have been shown to prolong progression-free survival (PFS) and overall survival (OS) [24–28] and are in fact approved for the treatment of solid cancer.

It has been previously demonstrated that the plasma levels of cytokines and angiogenic factors (CAFs) decrease after therapy in patients with cancer, and this may be relevant for treatment response and PFS [29–32]. Here, we demonstrate that high levels of CAFs are negative prognostic factors in patients with MM and seem to be predictive of relative benefit from therapy. Moreover, the stratification of patients based on CAF levels at diagnosis is useful to detect different risk groups for outcome and response to therapy.

## Methods

### Patients

Patient characteristics are reported in Table 1. This study has been carried out on MM patients enrolled in the multicenter clinical trial GIMEMA-MM0305, with the participation of 61 centers in Italy from May 2006 to January 2009. The study compared the combination bortezomib-melphalan-prednisone-thalidomide followed by maintenance with bortezomib-thalidomide (VMPT-VT) with bortezomib-melphalan-prednisone (VMP) administered for nine cycles without maintenance. The details and results of the trial have been published previously [33–35]. Clinical protocol and informed consent documents were approved by the participating local institution’s review boards, and the trial was undertaken in accordance with the International Conference on Harmonization Guidelines for Good Clinical Practice and the amended Declaration of Helsinki. Patients without MM or other tumors (patients with stage I arterial hypertension without organ damage and without other diseases) who gave their consent were used as controls.

**Table 1:** Patients’ characteristics

	Patients			Controls
	Total	VMP	VMPT	
*n*	124	53	71	54
Age	71 (56–85)	71 (60–85)	71 (56–85)	69 (54–88)
Sex (M/F)	58/66	24/29	34/37	24/30
Type of MM				NA
IgG (%)	71 (57.2)	31 (58.5)	46 (64.8)	
IgA (%)	28 (22.6)	12 (22.6)	14 (19.8)	
BJ (%)	25 (20.2)	10 (18.9)	11 (15.4)	
Stage (D&S)				NA
IIA (%)	14 (11.3)	5 (9.4)	9 (12.7)	
IIIA (%)	102 (82.3)	45 (84.9)	57 (80.3)	
IIIB (%)	8 (6.4)	3 (5.7)	5 (7)	
ISS stage				NA
1	26	8	18	
2	42	21	21	
3	21	6	15	
Missing data	35	18	17	
Cytogenetics				NA
High risk	28	11	17	
Standard risk	36	16	20	
Missing data	60	26	34	
Response				
CR (%)	47 (37.9)	14 (26.4)	33 (46.6)	
VGPR (%)	27 (21.8)	11 (20.8)	16 (22.6)	NA
PR (%)	38 (30.6)	19 (35.8)	19 (26.8)	
SD (%)	12 (9.7)	9 (17)	3 (4.2)	
Relapse (Y/N)	80/44	39/14	41/30	NA
Death (Y/N)	54/70	22/31	32/39	NA

*NA* not applicable

### Methods

Before starting the treatment, peripheral blood and bone marrow plasma (the initial 1 ml of bone marrow aspirate) samples were collected into EDTA-containing tubes. Both blood and bone marrow plasma samples from 124 of 511 patients enrolled in the study (24%) were available for analysis. Plasma was separated by centrifugation (2,000 rpm for 20 min at 4 °C) within 1 h from blood drawing and aliquoted into multiple cryovials. Plasma samples were stored at − 80 °C until use. Before analysis, plasma samples were thawed slowly in an ice bath and all analyses were done from a one-off thaw sample. CAFs were measured by using Q-Plex™ Array Human Angiogenesis Antigen (Quansys Biosciences, Logan, Utah) allowing the simultaneous quantification of the following factors: angiopoietin-2 (ANG-2), fibroblast growth factor-2 (FGF-2), hepatocyte growth factor (HGF), interleukin-8 (IL-8), platelet-derived growth factor-BB (PDGF-BB), tissue inhibitor of matrix metalloproteinase-1 and 2 (TIMP-1, TIMP-2), tumor necrosis factor-alpha (TNF-α), and vascular endothelial growth factor (VEGF), according to the manufacturer’s instructions. Secreted levels of CAFs were quantified through Q-View Software (Quansys Biosciences, Logan, Utah) in triplicate samples, and the mean results were used in biomarker analysis.

### Statistical analysis

In the first step, CAF levels were measured in the blood and bone marrow plasma samples of MM patients to assess whether significant differences could be detected in the two compartments by Student’s *t* test (*p* values less than 0.05 was considered significant). Since no significant differences between the two compartments were detected (see the “Results” section), subsequent analyses were carried out using plasma samples from the peripheral blood. With the Student *t* test, the CAF levels in the plasma samples of the MM patients were compared with those of controls.

In the second phase, plasma levels of CAFs were measured as an independent variable to predict binary response status (≥ VGPR vs < VGPR) by logistic regression analysis. CAF plasma levels were also correlated as a continuous variable with tumor response by linear regression and logarithmic transformation to normalize CAF values. The correlation between log-CAFs and tumor response was approximately linear. Selection of individual CAF markers from the screening phase was done on the basis of results of median cutoff, ROC curve estimation of cutoff, and logistic regression analysis between dichotomized tumor response and CAF plasma levels. We assessed the association between CAF plasma levels and progression-free survival (PFS) with the Cox proportional hazard model.

To establish whether plasma levels of any CAF might have prognostic or predictive significance, the Kaplan-Meier method was used to analyze PFS and OS. We used the Cox regression model to verify significant differences noted in Kaplan-Meier curves for both treatment groups between a high- and a low-CAF subgroup, defined by the respective median CAF plasma levels. Sensitivity analyses confirmed that median cutoff achieved the most significant segregation of clinical benefit. To assess the potential differential effects of baseline CAF concentrations between two treatment groups, a treatment versus CAF status interaction term was included in the Cox model analysis for PFS, with treatment group and CAF status as two additional independent variables. CAF plasma levels with a significant interaction value with treatment were regarded as predictive. A post hoc analysis was done to adjust for multiple testing of CAF markers with the Bonferroni test. Exploratory analyses included correlation between CAF plasma levels and Eastern Cooperative Oncology Group (ECOG) performance status, Durie & Salmon stage, International Staging System stage, Cytogenetic risk, and age and sex hierarchical clustering (unweighted pair group method with arithmetic mean, unweighted average, and Euclidean distance for similarity measure) to assess a multiple-CAF signature association with PFS or OS (Kaplan-Meier method for PFS and OS, and Cox regression models to assess differences). All statistical analyses were done with SPSS software.

## Results

Biological samples (blood and bone marrow) from 124 MM patients randomly assigned to receive VMPT-VT (53 pts) or VMP (71 pts) in the GIMEMA MM0305 phase III clinical trial and blood plasma samples from 54 control subjects were available for this study. Baseline demographic and disease characteristics are reported in Table 1.

We evaluated the concentration of ANG-2, FGF-2, HGF, IL-8, PDGF-BB, TIMP-1, TIMP-2, TNF-α, and VEGF that are the major cytokines involved in angiogenesis in MM and other cancers [36] and, as previously demonstrated [37] in MM patients, directly correlate with disease activity and increase with progression. Moreover, plasma levels of CAFs are directly related to disease response to therapy in hematologic and solid tumors [29–32].

**Fig. 1:**
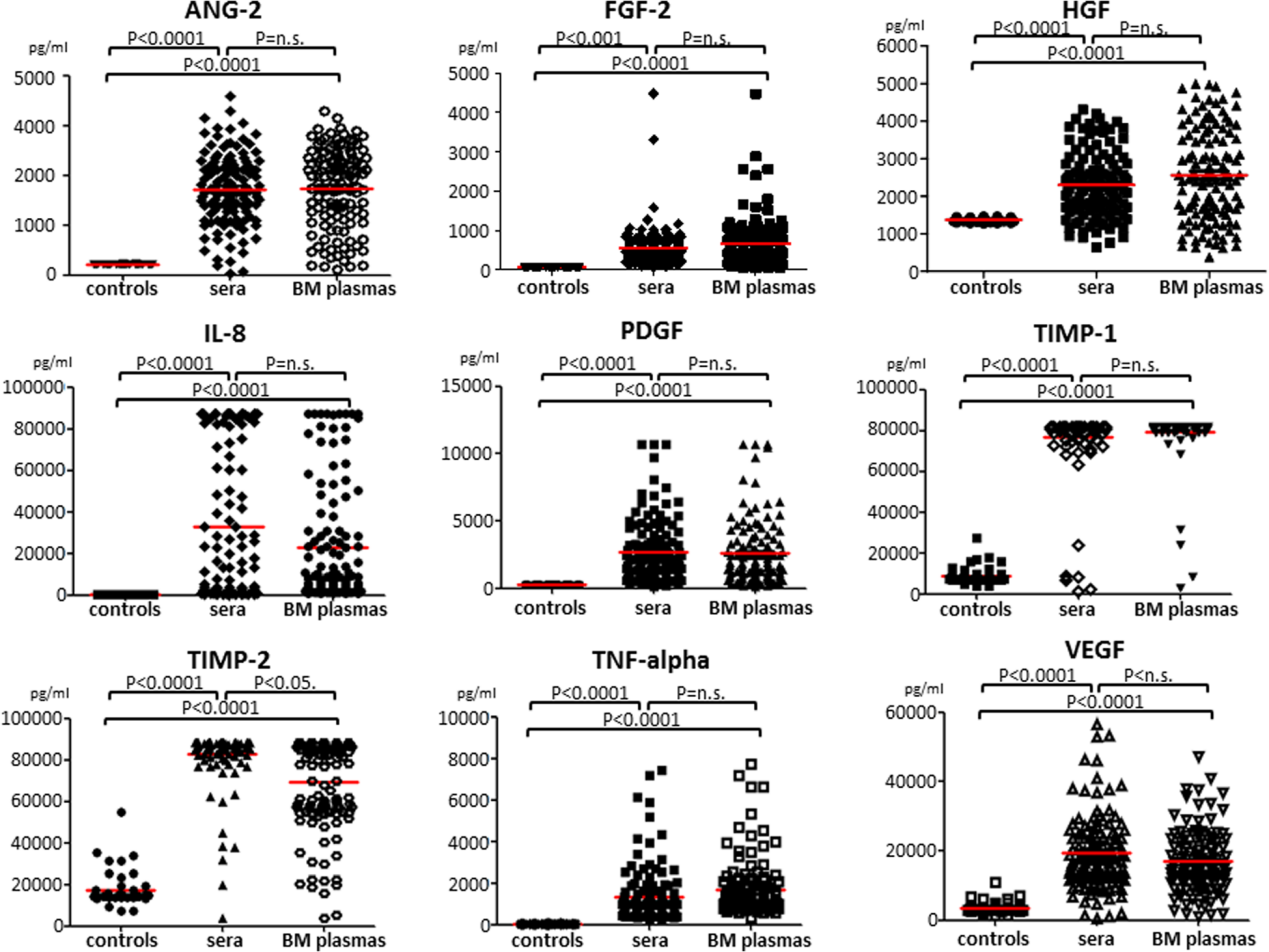
Analysis of the CAF levels in blood and bone marrow plasma samples of MM patients. No differences are evident in their concentration between peripheral blood and bone marrow samples. Significantly higher levels of CAFs are detected in blood and bone marrow samples of MM patients as compared with control subjects (*p* < 0.0001 for all cytokines)

**Fig. 2:**
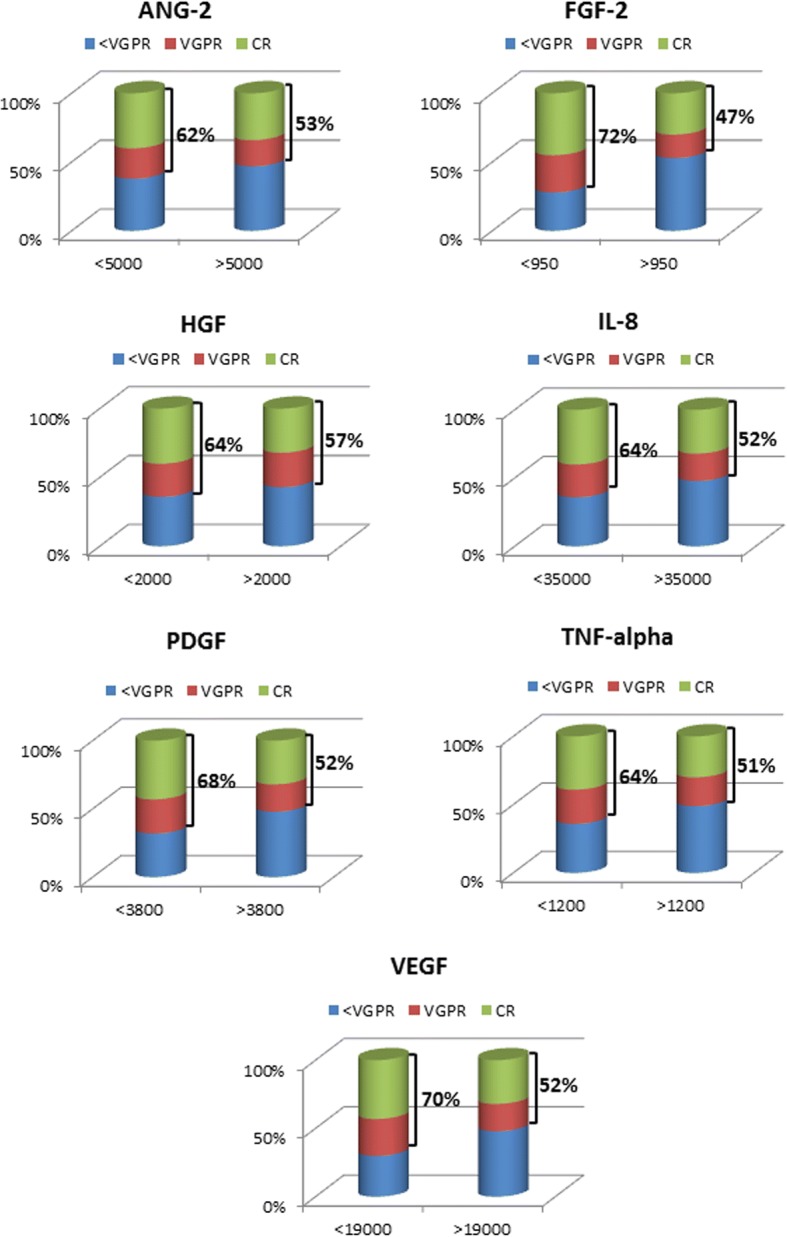
Response rate of MM patients based on CAF levels. The blood levels of CAFs significantly correlate with MM response to therapy. Lower levels of ANG-2 (*p* < 0.05), FGF-2 (*p* < 0.005), HGF (*p* < 0.05), IL-8 (*p* < 0.05), PDGF-BB (*p* < 0.005), TNF-α (*p* < 0.05), and VEGF (*p* < 0.005) are indicative of more profound response, VGPR or better, in all patients, with no evident differences between the two therapy regimens (VMPT-VT vs VMP: *p* = 0.1)

Our results showed that there were no differences in the levels of the studied CAFs between the peripheral blood and bone marrow plasma samples of MM patients (Fig. 1, Additional file 1: Table S1), indicating that the concentrations of circulating cytokines well reflect those of the bone marrow and could be used for all subsequent analyses. As expected, the plasma levels of CAFs in MM patients were significantly higher compared to controls (*p* < 0.0001 for all CAFs). In addition, the levels of CAFs were found to be significantly related to MM response to therapy (Fig. 2) with the exception of TIMP-1 and TIMP-2. More precisely, low levels of ANG-2 (*p* < 0.05), FGF-2 (*p* < 0.005), HGF (*p* < 0.05), IL-8 (*p* < 0.05), PDGF-BB (*p* < 0.005), TNF-α (*p* < 0.05), and VEGF (*p* < 0.005) were indicative of more profound response (very good partial response [VGPR] or better in all patients, with no evident differences between the two therapeutic regimens (VMPT-VT vs VMP: *p* = 0.1).

On the basis of ROC curve estimation of cutoff and logistic regression analysis between dichotomized tumor response and CAF plasma levels, a cutoff for each cytokine was established that could be used to discriminate the probability of response to therapy of MM patients with high sensitivity and specificity (Additional file 1: Table S2). Seventy-four patients had the best degree of tumor response (≥ VGPR) and were termed good responders, whereas 50 had the smallest degree of tumor response (< VGPR) and were termed poor responders. Among the seven CAFs that were shown to be significantly related to MM response to therapy (Fig. 2), low (relative to median) concentrations of ANG-2 (*p* < 0.0001), FGF-2 (*p* < 0.0001), HGF (*p* < 0.0001), IL-8 (*p* < 0.0001), PDGF-BB (*p* < 0.0001), TNF-α (*p* < 0.001), and VEGF (*p* < 0.0001) highly correlated with best response (Additional file 1: Table S2).

In terms of survival, PFS correlated with CAF levels as a dichotomous variable, and low levels of FGF-2 (*p* < 0.0001), HGF (*p* < 0.05), IL-8 (*p* < 0.05), TNF-α (*p* < 0.05), and VEGF (*p* < 0.005) were associated with better PFS. Instead, only low levels of FGF-2 (*p* < 0.001) and VEGF (*p* < 0.004) were associated with prolonged OS. Similar results were obtained when CAF levels were related to PFS and OS according to the combination therapy administered. A better PFS was in fact related to low levels of FGF-2 (*p* < 0.0001), HGF (*p* < 0.0001), TNF-α (*p* < 0.005), and VEGF (*p* < 0.005) in the VMP arm and FGF-2 (*p* < 0.005) and VEGF (*p* < 0.05) in the VMPT-VT arm. When OS was taken into consideration, only FGF-2 (*p* < 0.001 in the VMP arm, *p* < 0.005 in the VMPT-VT arm), and VEGF (*p* < 0.05 for both arms) were demonstrated (Additional file 1: Table S3).

**Fig. 3:**
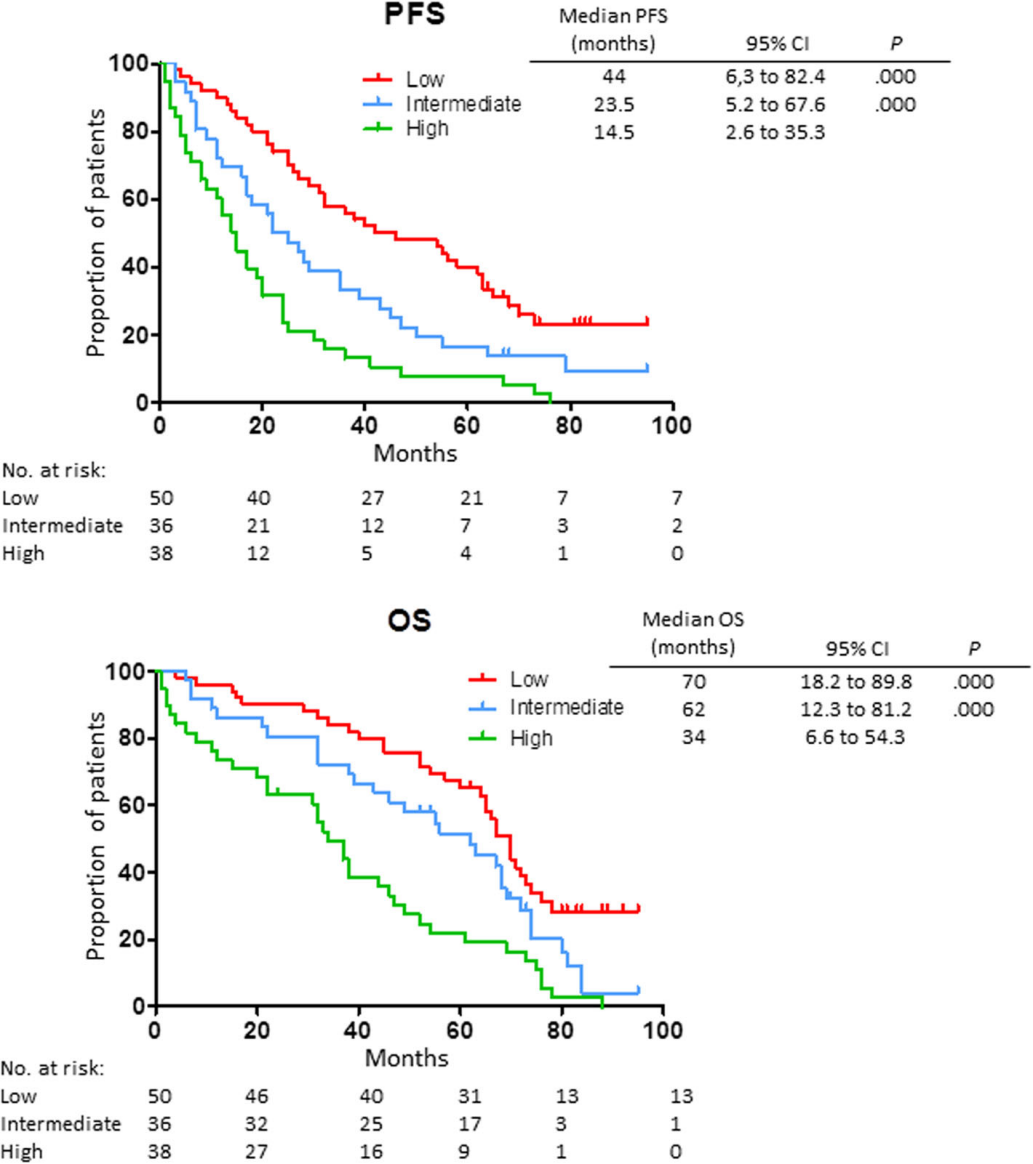
Progression-free and overall survival analysis in MM patients based on the peripheral blood plasma concentrations of FGF-2 and VEGF. The hierarchical clustering analysis of MM patients shows three distinct risk groups of patients based on the concentrations of FGF-2 and VEGF. *High risk*: patients who present both high FGF-2 and VEGF plasma levels showing a worse prognosis with significantly shorter PFS and OS; *intermediate risk*: patients who present high plasma levels in only one of the two cytokines; *low risk*: patients who show low blood levels in both angiogenic cytokines. Again, no evident differences between the two therapy regimens were detected

Hierarchical clustering analysis (Fig. 3) showed three distinct risk groups of patients, based on the concentrations of two CAF levels (FGF-2 and VEGF). Patients with elevation of both CAF levels had a worse prognosis with significantly shorter PFS and OS (high risk) compared with those with high level of only one (*p* < 0.0001, intermediate risk) and those of low blood levels of both CAFs (*p* < 0.0001, low risk) (Fig. 3). No significant differences were detected depending on the type of therapy received. Trying to construct a survival model, a three-stage system using FGF-2 and VEGF blood levels provided the highest statistically significant results (Table 2). Median survivals of the risk groups were as follows: low risk: PFS 44 months, OS 70 months; intermediate risk: PFS 23.5 months, OS 62 months; and high risk: PFS 14.5 months, OS 34 months (*p* < 0.0001 for differences). Patient numbers were well distributed across the three groups (low risk, 40%; intermediate risk, 29%; and high risk, 31%).

**Table 2:** Patient stratification based on CAF circulating levels

Risk group	Risk factors	Criteria	Median PFS (months)	Median OS (months)
Low	0	FGF-2 ≤ 950 pg/dL and VEGF ≤ 19,000 pg/dL	38	67
Intermediate	1	FGF-2 > 950 pg/dL or VEGF > 19,000 pg/dL	24	55
High	2	FGF-2 > 950 pg/dL and VEGF > 19,000 pg/dL	15	37

By multivariate analysis (Table 3), the other variables significantly associated with better outcome were age < 65 years in the intermediate- and high-risk groups (HR 0.61, CI 0.44–0.85, *p* = 0.0047 and HR 0.60, CI 0.38–0.89, *p* = 0.0391, respectively) for OS; age < 65 years and best response to induction therapy (≥ VGPR) only in the high-risk group (HR 0.68, CI 0.48–0.96, *p* = 0.0342) for PFS. Durie and Salmon (D&S) stage 1 or 2 impacted only on PFS for the high-risk group (HR 0.64, CI 0.49–0.89, *p* = 0.0169). No significant differences were demonstrated regarding both PFS and OS for sex, isotype of the M-component, renal failure, and type of induction therapy. The administration of maintenance therapy significantly impacted on PFS in all the risk groups (low: HR 0.70, CI 0.44–0.89, *p* = 0.0210; intermediate: HR 0.54, CI 0.38–0.80, *p* = 0.0008; high: HR 0.71, CI 0.56–0.92, *p* = 0.0249) and OS (low: HR 0.70, CI 0.48–0.88, *p* = 0.0210; intermediate: HR 0.74, CI 0.57–1.01, *p* = 0.0330; high: HR 0.52, CI 0.36–0.75, *p* = 0.0003).

**Table 3:** Multivariate analysis of risk stratification of patients based on the CAFs FGF-2 and VEGF

Variable	PFS						OS					
	Risk group						Risk group					
	Low		Intermediate		High		Low		Intermediate		High	
	HR (95 CI)	*p*	HR (95 CI)	*p*	HR (95 CI)	*p*	HR (95 CI)	*p*	HR (95 CI)	*p*	HR (95 CI)	*p*
Sex	0.82 (0.59–1.18)	0.3698	0.74 (0.59–1.11)	0.9645	0.86 (0.62–1.08)	0.3438	0.80 (0.57–1.05)	0.3698	0.96 (0.68–1.33)	0.6905	0.86 (0.59–1.16)	0.5713
Age (< 65 years)	0.96 (0.69–1.35)	0.7647	0.87 (0.55–1.20)	0.2027	0.73 (0.59–0.98)	*0.0455*	0.97 (0.71–1.32)	0.7647	0.61 (0.44–0.85)	*0.0047*	0.60 (0.38–0.89)	*0.0391*
Isotype	0.91 (0.56–1.32)	0.2957	0.91 (0.64–1.31)	0.6951	0.96 (0.68–1.33)	0.6419	0.82 (0.51–1.21)	0.2957	0.83 (0.57–1.15)	0.3627	0.98 (0.81–1.22)	0.8179
D&S stage	0.84 (0.60–1.16)	0.3218	0.82 (0.57–1.22)	0.3505	0.64 (0.49–0.89)	*0.0169*	0.83 (0.59–1.11)	0.3218	0.98 (0.67–1.44)	0.7874	0.74 (0.59–1.12)	0.0593
ISS stage	0.94 (0.66–1.29)	0.8163	0.91 (0.55–1.24)	0.2107	0.84 (0.58–1.28)	0.0619	0.79 (0.61–1.07)	0.1087	0.97 (0.66–1.34)	0.5221	0.87 (0.62–1.27)	0.0665
Cytogenetic risk	0.93 (0.64–1.28)	0.6358	0.87 (0.56–1.22)	0.3451	0.91 (0.55–1.33)	0.6670	0.86 (0.71–1.32)	0.2399	0.89 (0.69–1.41)	0.6133	0.92 (0.71–1.37)	0.6112
Renal failure	0.94 (0.61–1.25)	0.5866	0.92 (0.59–1.44)	0.6957	0.87 (0.60–1.21)	0.2410	0.92 (0.66–1.41)	0.5866	0.94 (0.71–1.35)	0.6108	0.97 (0.74–1.25)	0.8902
Induction therapy	0.84 (0.61–1.28)	0.3520	0.93 (0.66–1.34)	0.6838	0.88 (0.58–1.18)	0.3356	0.84 (0.57–1.19)	0.3520	0.93 (0.70–1.32)	0.6075	0.89 (0.66–1.28)	0.6015
Best response to induction therapy (≥ VGPR)	0.76 (0.59–1.18)	0.0984	0.68 (0.48–0.96)	*0.0342*	0.71 (0.55–1.08)	0.0533	0.74 (0.59–1.09)	0.0984	0.95 (0.69–1.31)	0.5031	0.88 (0.61–1.29)	0.0583
Maintenance	0.70 (0.44–0.89)	*0.0210*	0.54 (0.38–0.80)	*0.0008*	0.71 (0.56–0.92)	*0.0249*	0.70 (0.48–0.88)	*0.0210*	0.74 (0.57–1.01)	*0.0330*	0.52 (0.36–0.75)	*0.0003*

*p* values less than 0.05 was considered significant

As regards the cytogenetic risk, because many data relative to the cytogenetic characteristics of the patients were missing in the database of the trial, the statistical power of the relative analysis was low. For this reason, it is not possible to reach a correct conclusion on the value of CAFs stratification risk in correlation to this risk parameter.

## Discussion

The emergence of new treatment options for MM has extended the patients’ survival [5] and the need to prospectively identify those patients who are likely to benefit from a specific treatment and understand the mechanisms underlying therapeutic resistance. Several adverse prognostic factors have been identified in MM at diagnosis and before initiation of treatment [38, 39], including an advanced stage in the international staging system (ISS) based on plasma albumin [40]. The dual activity of the new and newest drugs active both on MM plasma cells and bone marrow stromal cells, and in particular on angiogenesis [41–43], obviously indicates that any new prognostic markers cannot ignore the angiogenesis aspect. Previous studies have indeed suggested that the plasma levels of CAFs might be used to identify prognostic and predictive markers in solid tumors [44–49] and are indicative of the response to antineoplastic therapy [29–32].

Seven CAFs of the nine evaluated (ANG-2, FGF-2, HGF, IL-8, PDGF-BB, TNF-α, and VEGF) initially emerged as being related to disease activity, but further testing showed that only FGF-2 and VEGF were significantly associated with PFS and OS and were therefore evaluated for prognostic stratification of patients. We also assessed whether determination of the studied CAFs could add prognostic information to D&S [50] and International Staging Systems (ISS) [40] or whether these systems were, in fact, predictive of benefit. D&S and ISS staging were associated with prognosis based on PFS in both groups; however, they were not strong prognostic parameters as FGF-2 and/or VEGF. Thus, the plasma levels of the two angiogenic cytokines provided prognostic and, more importantly, predictive value beyond that of standard clinical staging.

Our analyses of survival were based on assignment at initial randomization. High FGF-2 and VEGF plasma levels were negative prognostic markers and were associated with lower relative OS in both harms. The associations reported here with FGF-2 and VEGF were akin to previous studies [29–32], in which patients with the highest reduction of circulating CAF concentrations obtained the greatest benefit from anticancer therapy. However, other studies assessing the use of VEGF as a predictive marker of benefit from VEGF-targeted therapies in renal-cell carcinoma have yielded inconsistent results [51].

Previous studies have shown that groups of related angiogenic or inflammatory factors are often correlated [44, 46, 52]. Hierarchical clustering of six circulating CAFs showed a strong correlation among many of them, including osteopontin and VEGF [46, 52]. Patients defined by high concentrations of these CAF and inflammatory or immunomodulatory factors had a significantly worse prognosis, but derived a greater relative OS benefit from therapy. The circulating CAFs identified in these studies might themselves have important biological roles or might be markers for alternative pathways or mechanisms affecting treatment responses (e.g., hypoxia-induced factor-1α or NF-κB pathways). This association between factors suggests that common mechanisms might regulate their production. Studying a cohort of patients with metastatic renal-cell carcinoma, a classification of the disease based on the higher expression of angiogenic versus inflammatory circulating CAFs defined by a six-cytokine signature was established [46]. In that study, the angiogenic group derived greater benefit from sorafenib alone, whereas the other group benefited from the combination of sorafenib and IFN-α. In the present study, a similar correlation of treatment benefit with circulating CAF signature was observed in MM patients.

All the evidences provided by the studies on the role of microenvironment [3, 17], and particularly of the angiogenic process [23, 37] in myeloma progression as well as in cancer cell protection mediated by microenvironment components [37], indicated that the response to therapy is also related to the activity of anticancer drugs on tumor microenvironment [42, 43]. Therefore, the inhibition of cytokine production which mediates the interaction between cancer cells and their microenvironment represents one of the major goals of the modern therapeutic approaches. On these bases, the evaluation of CAF levels is indicative of the response to therapy and may represent a good indicator of refractoriness in cancer patients.

## Conclusion

Overall, our findings support the use of circulating CAF profiling to define biologically distinct subgroups of MM patients whose tumors have a greater angiogenic drive. As such, these patients might have a more aggressive disease course but are likely to derive relative benefit from inhibition of angiogenic pathways. Circulating CAF profiling might be particularly well suited for angiogenesis inhibitors and other drugs targeting the tumor microenvironment, in which both circulating host-derived and tumor-derived factors could affect response. Such an approach may have important advantages, including straightforward and relatively non-invasive sample collection, availability of robust analytical platforms, and the ability to monitor changes during treatment or disease progression, which can help identify markers of resistance.

The limitation of this study is that the results have been obtained in older patients not-eligible for ASCT, whereas the major strength of the study is the homogeneous stratification and longer follow-up of patients. Further studies on a greater cohort of subjects, including young patients eligible for ASCT before and after treatment, will be needed to evaluate the variation of CAFs after therapy, to study their power as an indicator of minimal residual disease and, then, of risk of relapse, and to evaluate the value of the risk stratification based on CAFs in the new drugs era, with the aim of definitively establish the value of this approach in the application of precision, personalized therapy for patients with MM.

## Additional file

Additional file 1: **Table S1.** CAF levels in serum and bone marrow plasma of MM patients. Table S2. Cutoff for serum levels of cytokines and angiogenic factors (CAFs). Table S3. PFS and OS correlation with CAF levels (PDF 460 kb)

## Abbreviations

ANG-2: Angiopoietin-2
CAFs: Cytokines and angiogenic factors
CI: Confidence interval
D&S: Durie and Salmon
ECOG: Eastern Cooperative Oncology Group
FGF-2: Fibroblast Growth Factor-2
HGF: Hepatocyte Growth Factor
HR: Hazard ratio
IL-8: Interleukin-8
ISS: International Staging System
MM: Multiple myeloma
OS: Overall Survival
PDGF-BB: Platelet-Derived Growth Factor-BB
PFS: Progression-Free Survival
TIMP: Tissue Inhibitor of Matrix Metalloproteinase
TNF-α: Tumor Necrosis Factor-alpha
VEGF: Vascular Endothelial Growth Factor
VGPR: Very Good Partial Response
VMP: Bortezomib-melphalan-prednisone
VMPT-VT: Bortezomib-melphalan-prednisone-thalidomide followed by maintenance with bortezomib-thalidomide
