# Supplementary material for: Prognostic or predictive value of circulating cytokines and angiogenic factors for initial treatment of multiple myeloma in the GIMEMA MM0305 randomized controlled trial
Source: J Hematol Oncol. 2019 Jan 9;12:4. doi: 10.1186/s13045-018-0691-4 (PMC6327520; doi:10.1186/s13045-018-0691-4)
Supplement: Supplementary file 1 — Table S1. CAF levels in serum and bone marrow plasma of MM patients. Table S2. Cutoff for serum levels of cytokines and angiogenic factors (CAFs). Table S3. PFS and OS correlation with CAF levels (PDF 460 kb) [file 13045_2018_691_MOESM1_ESM.pdf]

Table S1: CAF levels in serum and bone marrow plasma of MM patients.

**Total Patients**									
**Sera**									
	**ANG-2 (pg/ml)**	**FGF (pg/ml)**	**HGF (pg/ml)**	**IL8 (pg/ml)**	**PDGF (pg/ml)**	**TIMP-1 (pg/ml)**	**TIMP-2 (pg/ml)**	**TNFalfa (pg/ml)**	**VEGF (pg/ml)**
**Number**	124	124	124	124	124	124	124	124	124
**Mean±SD (range)**	264939±20833 (13405-1072635)	15699±2857 (2259-344121)	66817±4080 (9824-374475)	818085±80798 (6805-2177235)	110129±4071 (2112-229504)	769222±14283 (11792-817657)	415173±6061 (17925-442738)	42728±7779 (6404-671451)	619446±30566 (15699-1815946)
**BM plasmas**									
	**ANG-2 (pg/ml)**	**FGF (pg/ml)**	**HGF (pg/ml)**	**IL8 (pg/ml)**	**PDGF (pg/ml)**	**TIMP-1 (pg/ml)**	**TIMP-2 (pg/ml)**	**TNFalfa (pg/ml)**	**VEGF (pg/ml)**
**Number**	124	124	124	124	124	124	124	124	124
**Mean±SD (range)**	256710±21301 (13405-1072261)	67013±15318 (8593-1041861)	150319±12424 (9824-493076)	567520±63861 (17303-2177104)	111452±4567 (7489-211233)	791141±10579 (30323-817657)	346229±9790 (17925-442738)	41955±3068 (7792-192997)	540654±25225 (33458-1510377)
**V-MP**									
**Sera**									
**Number**	53	53	53	53	53	53	53	53	53
**Mean±SD (range)**	255827±26922 (32604-971297)	12108±8489 (2673-31663)	71901±7824 (17809-374475)	878205±130492 (6805-2177235)	106373±5863 (2112-202310)	790400±15528 (11792-817657)	424626±5966 (159984-442738)	47243±12964 (8423-658833)	679089±43561 (15699-1815946)
**BM plasmas**									
**Number**	53	53	53	53	53	53	53	53	53
**Mean±SD (range)**	264260±37738 (13405-1072261)	91784±32738 (8608-1041861)	155366±19330 (9824-493076)	547656±100662 (17303-2177104)	116460±7474 (11100-211233)	785809±17588 (82385-817657)	345475±15051 (17925-442738)	39519±3474 (14827-132839)	567349±43133 (33458-1309659)
**V-MPT**									
**Sera**									
**Number**	71	71	71	71	71	71	71	71	71
**Mean±SD (range)**	271741±30471 (13405-1272561)	18378±4941 (2259-344121)	63022±4083 (9824-159319)	773206±102589 (9064-2475784)	112932±5616 (4061-229504)	753413±22001 (24447-817657)	408116±9558 (17925-442738)	39357±9597 (6404-671451)	574925±41817 (33458-1254471)
**BM plasmas**									
**Number**	71	71	71	71	71	71	71	71	71
**Mean±SD (range)**	251074±24528 (19810-1125635)	48475±10696 (8593-495306)	146551±16310 (11880-493076)	582348±83001 (18034-2365982)	107714±5705 (7489-181369)	795121±13091 (30323-817657)	346791±12980 (26279-442738)	43773±4700 (7792-192997)	520727±30129 (97211-1639584)

Table S2: Cut-off for serum levels of cytokines and angiogenic factors (CAFs)

CAFs	Cut-off (pg/ml)	AUC	St. Error	95% CI	Sensitivity%	95% CI	Specificity%	95% CI	Likelihood ratio	P value
**Ang-2**	5000	0,8028	0,03477	0.7346 to 0.8710	66,15	53.35% to 77.43%	65,88	54.80% to 75.82%	1,94	<0.0001
**FGF-2**	950	0,9814	0,008262	0.9652 to 0.9976	93,85	84.99% to 98.30%	94,12	86.80% to 98.06%	15,95	<0.0001
**HGF**	2000	0,8260	0,03350	0.7603 to 0.8916	76,92	64.81% to 86.47%	77,65	67.31% to 85.97%	3,44	<0.0001
**IL-8**	35000	0,8338	0,03313	0.7688 to 0.8987	76,92	64.81% to 86.47%	75,29	64.75% to 84.01%	3,11	<0.0001
**PDGF**	3800	0,7449	0,04002	0.6664 to 0.8233	67,69	54.95% to 78.77%	68,24	57.24% to 77.92%	2,13	<0.0001
**TNF-alpha**	1200	0,7962	0,03656	0.7245 to 0.8679	73,85	61.46% to 83.97%	74,12	63.48% to 83.01%	2,85	<0.001
**VEGF**	19000	0,8450	0,03108	0.7841 to 0.9059	75,38	63.13% to 85.23%	75,29	64.75% to 84.01%	3,05	<0.0001

Table S3. PFS and OS correlation with CAF levels

**Cytokine**	**ANG-2 <5000**	**ANG-2 >5000**	**p**	**FGF-2 <950**	**FGF-2 >950**	**p**	**HGF <2000**	**HGF >2000**	**p**	**IL-8 <35000**	**IL-8 >35000**	**p**	**PDGF-BB <3800**	**PDGF-BB >3800**	**p**	**TNF-α <1200**	**TNF-α >1200**	**p**	**VEGF <19000**	**VEGF <19000**	**p**
**All pts**																					
Mean PFS months	32	24.5	.5	36.5	15	**.0001**	32	18.5	**.01**	29	20.5	**.01**	27	24	.8	33	19.5	**.03**	29	21	**.001**
Mean OS months	61	50.5	.1	67	38	**.0006**	63	49	.1	63	49	.3	63	49	.1	62	44	.3	65	45	**.004**
**VMP**																					
Mean PFS months	29	22	.1	35	16	**.0001**	31	18	**.0001**	27	20	.3	25	20	.9	32.5	18	**.003**	25	20.5	**.04**
Mean OS months	62	46	.7	66	38	**.006**	64	44	.09	63	44	.06	63	46	.06	65	38	.07	63	45	**.04**
**VMPT-VT**																					
Mean PFS months	32	29	.6	40	15	**.004**	32	22	.8	32	21	.5	32	29	.7	35	22	.6	32	23	**.02**
Mean OS months	61	62	.3	67	40	**.04**	60	62	.8	61	62	.5	65	60	.8	60	62	.9	65	49	**.03**
